# P-579. Comparison of Extended Spectrum Beta-Lactamase Producing *Escherichia coli* Clonotypes Colonizing and Infecting Humans, Dogs and Cattle in Wisconsin Reveals Greatest Similarity Between Humans and Dogs

**DOI:** 10.1093/ofid/ofaf695.793

**Published:** 2026-01-11

**Authors:** Laurel Legenza, Veronika L Tchesnokova, Evgeni V Sokurenko, Joshua Petrie, Thomas R Fritsche

**Affiliations:** University of Wisconsin-Madison School of Pharmacy, Madison, Wisconsin; University of Washington, Seattle, Washington; University of Washington, Seattle, Washington; Marshfield Clinic Research Institute, Marshfield, Wisconsin; Marshfield Clinic Health System, Marshfield, Wisconsin

## Abstract

**Background:**

Mitigation of antimicrobial resistance in human and veterinary medicine relies on diagnostics, surveillance, and stewardship. Previously, we reported the prevalence of third-generation cephem-resistant (3GCR) *Escherichia coli* colonizing humans (3.7%), dogs (13.1%), and cattle (51.5%) in our service area (Ortiz 2022). Here, we present new data using a novel clonotyping method to characterize clonal composition of 3GCR *E. coli* among these hosts.

**Methods:**

Previously collected 3GCR *E. coli* isolates colonizing or infecting humans and dogs, and colonizing cattle in Wisconsin, were analyzed. Clonotyping was performed using a rapid method that detects 7 single nucleotide polymorphisms within two genes (fumC and fimH), yielding results comparable to multilocus sequence typing (Tchesnokova 2016). Only major clonotypes, comprising ≥2% of isolates per host, were included. Clonotype 561, corresponding to the pandemic multidrug-resistant ST131-H30 subclone, was excluded to avoid biasing the human clonal composition. Linear regression was used to compare clonotypes' composition and abundance between host species, and with a national dataset of 474 uropathogenic 3GCR *E. coli* isolates from humans (courtesy of Prof. E. Sokurenko). Comparisons were made by host species, and by source (Wisconsin vs. national).

**Results:**

A total of 447 Wisconsin 3GCR isolates were analyzed (human n=220; dog n=140; cattle n=87). Linear regression analysis showed a strong correlation between human and dog clonotypes, and vice versa (R²=0.924, 0.903). Human and dog clonotypes correlated poorly with cattle clonotypes (R²=0.268, 0.353), and cattle clonotypes correlated poorly with those from humans and dogs (R²=0.365, 0.301). Wisconsin human clonotypes correlated strongly with the national human dataset (including clonotype 561; R²=0.977, 0.966). Dogs showed moderate correlation (R²=0.701, 0.674), whereas cattle correlated poorly with the national dataset (R²=0.208, 0.260).

**Conclusion:**

While cattle harbor a larger reservoir of transferable resistance genes, the clonal composition of 3GCR *E. coli* in this collection shows significantly greater similarity between humans and dogs than between humans and cattle, or between dogs and cattle.

**Disclosures:**

Joshua Petrie, PhD, CSL Seqirus: Advisor/Consultant|CSL Seqirus: Grant/Research Support|ModernaTX: Grant/Research Support

